# Population-based consultation patterns in patients with shoulder pain diagnoses

**DOI:** 10.1186/1471-2474-13-238

**Published:** 2012-11-29

**Authors:** Eva Tekavec, Anna Jöud, Ralf Rittner, Zoli Mikoczy, Catarina Nordander, Ingemar F Petersson, Martin Englund

**Affiliations:** 1 Occupational and Environmental Medicine, Laboratory Medicine Lund, Lund University, Lund, SE-221 85, Sweden; 2 Department of Orthopedics, Clinical Sciences Lund, Lund University, Lund, SE-221 85, Sweden; 3 Clinical Epidemiology Research & Training Unit, Boston University School of Medicine, Boston, MA, 02118, USA

## Abstract

**Background:**

To assess the annual consultation prevalence and new onset consultation rate for doctor-diagnosed shoulder pain conditions.

**Methods:**

We identified all residents in the southernmost county in Sweden who received a shoulder pain diagnosis during 2006 (ICD-10 code M75). In subjects who did not consult due to such disorders during 2004 and 2005, we estimated the new onset consultation rate. The distribution of specific shoulder conditions and the length of the period of repeated consultation were calculated.

**Results:**

Annual consultation prevalence was 103/10 000 women and 98/10 000 men. New onset consultation rate was 80/10 000 women (peak in age 50–59 at 129/10 000) and 74/10 000 men (peak in age 60–69 at 116/10 000). About one fifth of both genders continued to consult more than three months after initial presentation, but only a few percent beyond two years. Rotator cuff - and impingement syndromes were the most frequent diagnoses.

**Conclusion:**

The annual consultation prevalence for shoulder pain conditions (1%) was similar in women and men, and about two thirds of patients consulted a doctor only once. Impingement and rotator cuff syndromes were the most frequent diagnoses.

## Background

Musculoskeletal disorders are common in industrial countries [1,2] and bring enormous costs to the society. In the European Union the cost of treatment and lost productivity is estimated to 0.5-2% of the gross domestic product [3,4] and in 2000 in the United States, the direct cost for treatment of shoulder dysfunction was estimated to $7 billion [5]. There was approximately 3.75 million working days per year lost in the United Kingdom (2008–2009) due to musculoskeletal problems [6]. In Sweden musculoskeletal problems represent about one third of all sick-leave and make up for the majority of all long-term sick leave [7,8]. Besides the economical burden, shoulder pain causes great individual harm and influences both work and private life.

The shoulder has been reported as the third most common site of musculoskeletal pain after low back pain [9] and knee pain [10]. In the general population a point prevalence of 7-26%, and one-year prevalence of 7-47% of self-reported shoulder pain are reported [3,11-14] while the annual incidence is estimated to be around 1-2% [15]. Among patients in primary care the annual consultation prevalence has been estimated to range from 2-10% of the population [15-17] and incidence from 11-30/1000 person-years [14-16,18]. Besides individual risk factors, such as age, arthritis, obesity, diabetes and thyroid disease [3,19-21], a strong relationship between working conditions and shoulder disorders has been reported in several studies [11,22,23]. Also, regional pain in the shoulder can evolve into more generalized pain syndromes [24]. The gender impact on shoulder disorders is not clear and is a topic of discussion [25,26].

Considering the tremendous burden of shoulder conditions on society, an up-to-date estimate of the burden on the health care system is important. Further, the prognosis, in terms of repeated consultations, is also of interest. Many previous studies are based on questionnaires often including mild and transient self-reported symptoms [15]. However, only about 20-50% of subjects with shoulder symptoms are likely to ever consult a doctor for their problem [15,16]. To assess such an estimate, registers of health care may be used. Since shoulder complaints are typically handled in primary care, information on both in-patient and out-patient data is required. The Skåne Health Care Register (SHCR), which covers the population in the southernmost part of Sweden, encompasses such data with diagnostic codes by medical doctors. Enabled by SHCR, the aim of this study was to gain new insights of the consultation patterns of doctor-diagnosed shoulder disorders by age and gender.

## Methods

We used population-based health care register data from Skåne County, the most southern county of Sweden (population Dec 31, 2005: women 593,569; men 575,895; total 1,169,464). The study was approved by Lund University institutional review board.

### The Swedish population register

In Sweden all residents are registered with a 10-digit personal identification number. Births, deaths and change of residential address are all registered in the Swedish population register.

### The Swedish health care system

Both public and private health care providers have the same tax-based financing system. Apart from a small patient co-pay (up to maximum approx. $100 per year) all residents are entitled to free health care, and this care can be provided by either a private and/or a public health care provider. Both are easily accessed by any resident (cost is the same). By law all health care provided has to be registered.

### Skåne health care register (SHCR)

Information entered in the register includes the patient’s personal identification number, health care provider, date, and type of consultation (clinic visit, telephone contact etc.). Further, diagnostic codes according to International Classification of Diseases and Related Health Problems (ICD) 10 system are forwarded to the SHCR for all consultations to a doctor in public health care. The register has recently been utilised for clinical epidemiologic studies to determine the prevalence of rheumatoid arthritis and spondyloarthritis, showing high validity of the diagnostic coding [27,28].

### The ICD-system

ICD-10 is a further development of previous ICD systems of classification that have been used since the 1850 s. ICD-10 has been used in the Skåne County in Sweden since 1998. Shoulder lesions are coded M75 (M chapter covering “Diseases of the musculoskeletal system and connective tissue”). By adding a digit in the fourth position, doctors may specify type of shoulder diagnosis (Table 1) [29]. In primary care the diagnostic code M75.9P is used for shoulder problems in general and can thus include any of the specific M75 diagnoses (M75.0-M75.9).

**Table 1:** ICD-10 codes for shoulder pain diagnoses, annual consultation prevalence and new onset consultation rate for adult women and men in 2006 in southern Sweden

**ICD-10 code**	**Shoulder pain diagnosis**	**Annual consultation prevalence**		**New onset consultation rate**	
		**Women**	**Men**	**Women**	**Men**
		**per 10 000 (N)**	**per 10 000 (N)**	**per 10 000 (N)**	**per 10 000 (N)**
M75.0	Adhesive capsulitis	15 (477)	9 (263)	12 (368)	6 (190)
M75.1	Rotator cuff syndrome	30 (978)	32 (967)	25 (764)	24 (722)
M75.2	Bicupital tendinitis	5 (153)	5 (144)	4 (133)	4 (124)
M75.3	Tendinitis with calcification	6 (186)	4 (121)	5 (152)	3 (98)
M75.4	Impingement syndrome	25 (797)	30 (914)	17 (525)	21 (608)
M75.5	Bursitis of the shoulder	5 (163)	4 (109)	4 (128)	3 (93)
M75.8	Other, specific disorder in the shoulder	0.2 (6)	0.1 (4)	0.2 (5)	0.1 (4)
M75.9 + P	Other, unspecific disorder in the shoulder	17 (535)	15 (451)	13 (403)	12 (349)
M75	Total	102 (3295)	98 (2973)	80 (2478)	74 (2188)

### Definition of diagnoses

For patients with more than one consultation with a shoulder pain diagnosis during 2006, we considered the last diagnosis set by a specialist to be the most valid. For patients who had only consulted in primary care, we used the last diagnosis in that setting.

#### Subclasses of diagnoses

As the ICD-10 system does not give specific criteria for separate diagnoses, and these are overlapping concerning tissue specificity, we collapsed the shoulder pain diagnoses into three major classes based on the ICD-10 coding: 1; Adhesive capsulitis (M75.0), 2; Tendinitis, bursitis and impingement of the shoulder (M75.1-5) and 3; Unspecified shoulder pain diagnoses (M75.8, M75.9 and M75.9 P).

### Study cohort

We extracted data from the SHCR for all individuals who had been diagnosed with at least one shoulder pain diagnosis during 2006. We linked SHCR data with the population register in order to include only subjects who were residents in the Skåne County by December 31, 2005. We choose to primarily focus on individuals 20 years of age or older in our analyses, but in the figures data on children and adolescents are also presented.

### Statistics

#### Annual consultation prevalence

The annual consultation prevalence was calculated by dividing the number of unique Skåne residents who had been diagnosed with a shoulder pain diagnosis at least once during 2006 by the total adult population of Skåne by the last of December 2005. According to the SHCR in 2006, 30% of all outpatient doctor consultations in the Skåne County were within the private sector (not captured with diagnosis in the SHRC). Therefore, to compensate for loss of patients only seen by private practitioners, the population (the denominator), was reduced by 30%. We present results for women and men separately using 10-year age strata.

#### New onset consultation rate

Individuals with a new onset shoulder pain diagnosis were defined as patients with a shoulder pain diagnosis in 2006 but no such diagnosis in neither 2004 nor 2005. As the denominator we used the same as above (the total population in Skåne by the last of Dec 2005 reduced by 30%), but with the restriction that individuals also had to be resident in Skåne the two-year period before diagnosis, 2004–2005. We also evaluated the differences between men and women in occurrence of subclasses of diagnoses, by calculating the new onset consultation rate ratio and their 95% confidence interval (95% CI).

#### Length of consultation period

To evaluate the prognosis after new onset shoulder pain diagnosis, measured as the time period of repeat doctor consultations for shoulder pain in each patient, we extracted health care register data also for the three following calendar years (2007 until 2009). We defined the consultation period as the time between the first and last doctor consultation for a shoulder pain diagnosis within this time frame. In order to be counted, we required a repeat consultation to have occurred within 1 year from the prior consultation. We present results as the proportion of patients who only consulted once, and the proportion that was still consulting for a shoulder pain at more than 3 months, 6 months, 1 year, and at more than 2 years from their new onset consultation in 2006. Only individuals who were resident in Skåne from 2004 until the end of 2009 were included.

## Results

### Characteristics of the study cohort

In 2006, 3295 women age 58 years and 2973 men, age 56 years consulted for a shoulder diagnosis in the ICD-10 M75 group. There were 477 women and 263 men in the group of adhesive capsulitis (M75.0), 2277 women and 2255 men in the group of tendinitis, bursitis and impingement of the shoulder (M75.1-5) and finally 541 women and 455 men in the unspecified group (M75.8, M75.9 and M75.9 P).

### Annual consultation prevalence

**Figure 1:**
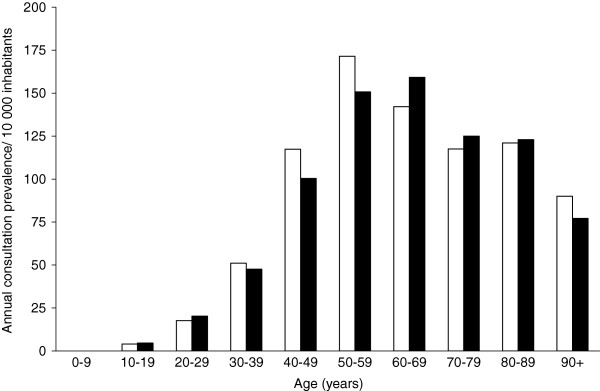
**Annual consultation prevalence due to shoulder pain diagnoses in southern Sweden.** Women = white bars. Men = black bars.

Among adults, the 2006 consultation prevalence (period prevalence) for a shoulder pain diagnosis was 103/10 000 women and 98/10 000 men. The consultation prevalence increased by age for both genders, reaching a peak in women 50 to 59 years of age (171/10 000 per year) and in men at 60 to 69 years of age (160/10 000 per year) before leveling off (Table 1, Figure 1).

### New onset consultation rate

**Figure 2:**
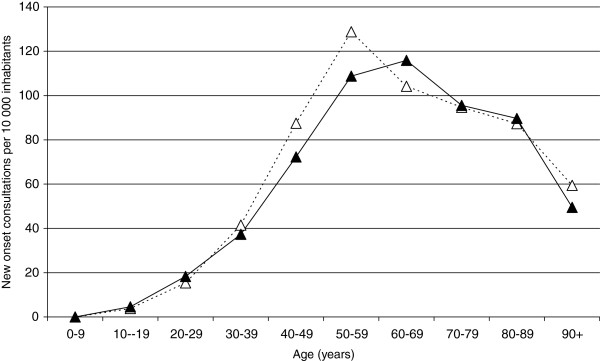
**New onset consultation rate due to shoulder pain diagnoses in southern Sweden.** Women = white symbols. Men = black symbols.

During 2006, the new onset consultation rate among adults was 80/10 000 for women and 74/10 000 for men (Table 1). The consultation rate increased by age for both genders, reaching a peak for women 50 to 59 years of age (129/10 000 per year) and for men 60 to 69 years of age (116/10 000 per year), and then leveling off (Figure 2).

### Proportion of subclasses of shoulder pain diagnoses

For annual consultation prevalence as well as for new onset rate, the group with tendinitis, bursitis and impingement dominated, representing 69% of shoulder pain diagnoses in women and 75% in men (Table 1). Women had a higher new onset consultation rate of adhesive capsulitis than men, rate ratio 1.71 (95% CI 1.45-2.02). On the other hand, men had a higher new onset consultation rate for impingement syndrome than women, rate ratio 1.31 (95% CI 1.18-1.45).

### Length of the consultation period

**Figure 3:**
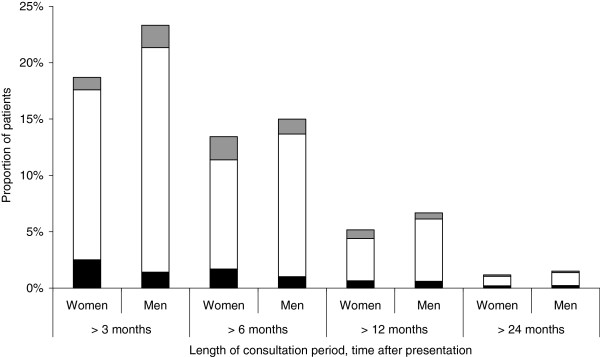
**Proportion of adults with new onset consultation of a shoulder pain diagnosis that continued to see a doctor for this condition beyond 3, 6, 12 and 24 months, respectively.** Shoulder pain diagnoses are divided into three subgroups; adhesive capsulitis (grey); tendonitis/bursitis and impingement syndrome (white) and unspecified (black).

Among new onset consultations in 2006, about two thirds of men as well as women consulted a doctor and were given a shoulder pain diagnosis only once (data not shown). About one fifth for both genders (19% women and 23% men) still consulted after more than 3 months (Figure 3). Only a minority (1-2%), of both genders kept consulting beyond two years. The distribution of the different shoulder pain subclasses was similar in those with repeat clinic visits as in those with a single visit (Figure 3).

## Discussion

The annual consultation prevalence for shoulder pain diagnoses was about 1% of the adult population and similar in both women and men. The new onset consultation rate was 80/10 000 per year for women and 74/10 000 per year for men. The consultations for shoulder pain diagnoses increased by age and peaked for women at age 50–59 and for men at age 60–69, where the incidence was much higher for both genders, compared to the whole working age group. Our study shows a somewhat lower annual consultation prevalence and new onset consultation rate leading to consultation in general practice than previous studies from UK and the Netherlands that suggest a prevalence and incidence of 236-480/10 000 and 112-300/10 000 [14-18,30] respectively. Possible explanations could include, but not be limited to, differences in study design (e.g. case criteria), health care systems and/or the more frequent use of symptomatic codes. Linsell *et al.* for example, also included acute trauma diagnoses and, Bot *et al.* included a general diagnostic code for “shoulder complaints”. Dorrestijn *et al.* report that in only 14% a specific diagnosis was recoded [30]. When taking this into account, the occurrence of shoulder pain conditions in Sweden seems to be about similar as in the United Kingdom and Holland.

Many shoulder pain diagnoses are associated with increasing age and a general degeneration of the body. In accordance with previous studies, new onset consultation rate increased by age, showing a peak in age 50–60 [14-16]. In fact the shape of the curve for new onset consultation rate had a similar shape in the report from Bot *et al.* compared to our Figure 2, though we present results for women and men separately. There was an increase in the new onset consultation rate after entering working life and a leveling off after retirement age (in Sweden at age 65.

In concordance with previous studies, tendinitis, bursitis and impingement syndrome, were the most frequent group of diagnoses for both women and men [5,18,22] Van der Wind *et al.* report that “subacromial syndrome” was the most frequently diagnosed disorder , in particular rotator cuff tendonitis (29%) [18].

Most patients with a new onset shoulder pain diagnosis consulted a doctor only once, which is in accordance with previous studies where 50% of new consulters only visited their GP once [15,16] and only few continued consulting beyond two years, in particularly the elderly [15]. We chose to exclude subjects with a consultation free period of more than one year although some of these may still have ongoing pain, as well as being at higher risk for relapses. Not consulting does naturally not equal being free of symptoms. A history of persistent shoulder complaints has shown to be a good predictor for slow recovery over time or recurrence of complaints [31,32] and about half of patients in primary care still reported complaints after 12 months [33] or experienced recurrent episodes during an 18-month follow up [34].

Some previous studies have shown that women have a higher risk of upper extremity disorders, compared to men (age and occupation adjusted) [25,35,36]. A systematic review of several databases found that women more often than men had neck-shoulder complaints with job tasks involving the same arm postures as men, while for job performances with hand-arm vibration men dominated [26]. Some studies indicate that gender differences in response to physical work exposure may reflect gender segregation in work and differences in pinch and lifting capacity [37,38]. Our study did not show any substantial differences between new onset consultation rate of shoulder pain diagnoses among women and men at large, which is in accordance with the result from Linsell *et al*[15]. Other studies did find a higher incidence among women [14,17,18]. Our results showed, however, that women reached their peak of annual consultation prevalence as well as new onset consultation rate earlier in their lifespan than did men. Notably, within the group of shoulder pain diagnoses, men consulted more frequently due to impingement syndrome compared to women, while adhesive capsulitis, which is not typically associated with occupational conditions [39], was more often diagnosed among women. Possibly the patients sex could influence the choice of diagnosis, so that it is more likely that the doctor will give a diagnosis of frozen shoulder to a woman than a man, since it is known that this condition is more common among women [40].

### Methodological considerations

The results of this study are limited to the ICD-10 diagnostic group M75, which does not constitute a fully comprehensive group on all shoulder pain diagnoses. For example Myalgia of the shoulder/upper arm would be coded M79.1B, Ligamentous Instability of the Shoulder M24.2B, Post traumatic Arthrosis of the Glenohumeral joint M19.1B, Recurrent dislocation and subluxation of the Shoulder joint M24.4B and Pain in Shoulder joint Unspecified M25.5B. However, these rather specific diagnoses are relatively uncommon in the register (we would only have identified an additional 359 subjects, an increase of the cohort size by 5.7%). Further, some disorders would be included in other more general or unspecified diagnoses, e.g., generalised pain syndrome or myalgia (without site code). It is however plausible that some such disorders, especially myalgia, have been diagnosed as unspecific (M75.9 + P), and thus included. Unfortunately, we are unable to ascertain the proportion of the patients diagnosed with unspecific myalgia that truly consulted with pain localized to the shoulder. Shoulder lesions diagnosed due to acute trauma such as fractures, dislocations, and contusions have not been included. In all we suggest the consultation rates in the present report should be considered as conservative estimates.

We have chosen to divide the shoulder pain diagnoses into three major groups. Adhesive capsulitis might have a different aetiology than tendinitis, bursitis and impingement syndrome, for which ergonomic conditions at work may be important [41]. One study shows that at 6 months non-recovery was reported to be more frequent in the group of patients with a non-specific diagnosis compared to a more specific one [38]. Unspecific shoulder pain diagnoses should therefore be looked upon separately. Most of these are in fact M75.9 P, i.e., any shoulder pain diagnosis set in primary care.

Further, since the shoulder has a complex anatomy and function tendinitis and bursitis around the shoulder could be difficult to differentiate, many symptoms overlap and specific shoulder pain diagnoses tend to cluster within the same individual. A consensus on the terminology and diagnostic classification system is essential for comparable results between studies [42-44]. Several attempts to agree on consensual case definitions and classificatory schemes have been made [44-47]. But in a systematic review no two of 27 schemes were identical [48].

Moreover, interobserver variation in physical examination and diagnostic interpretation may be a problem [44-46]. On the other hand there is some evidence that in population-based aetiological research and surveillance, simple case definitions are sufficient [47]. One way to possibly increase the accuracy of the diagnoses would be to review the medical records. However, shoulder diagnosis criteria tests are rather unspecific and difficult to evaluate and the retrieval and review of medical records ethically complex and time consuming. Hence, we have chosen not to make such an attempt of validation, instead we largely report data on the shoulder pain diagnoses collapsed together.

An important limitation of this study is that we could only accesses diagnostic codes from public health care. Hence, we needed to adjust our estimates for the missing data which may introduce bias. This would be true for example if younger subjects had a higher tendency to consult private care than elderly ones. However, among patients who consulted privately in 2006, 66% were aged 20–69 years. The corresponding proportion among those who consulted publicly was 60%. Hence, the subjects who consulted a private practitioner were only slightly younger than individuals who consulted public care. Further, importantly, in Sweden there is a large overlap of private and public consultations by the patients. For example, during 2006, the majority (67%) of subjects who had consulted privately at least once had also consulted public care the same year. This is because referrals in between public and private care are common. Both systems have the same basic financing and co-pay.

Moreover, as the SHCR does not cover occupational health services provided by employers, individuals with ongoing employment who have only consulted an occupational health physician due to their shoulder pain would not be identified. Therefore, the figures for working age individuals might be somewhat underestimated. However, in all, we expect any socioeconomic selection bias not to be as dramatic as in other health care systems.

## Conclusions

Prevalence and new onset consultation rate were about equal in women and men. The rates increased from entering working life and decreased from retirement age. Shoulder pain diagnoses with a possible relationship to adverse ergonomics were the most frequent.

## Abbreviations

SHCR: Skåne Health Care Register; ICD-10: International Statistical Classification of Diseases and Related Health Problems.

## Competing interests

The authors declare that they have no competing interests.

## Authors’ contribution

All authors have made substantial contributions to (1) the conception and design of the study, or acquisition of data, or analysis and interpretation of data, (2) drafting the article or revising it critically for important intellectual content, (3) final approval of the version to be submitted. Specific contributions are: 1. The conception and design of the study: ET, CN, AJ, IP, ME; 2. Acquisition of data: AJ; 3. Analysis of data: ET, RR, ZM, AJ; 4. Interpretation of results: all authors; 5. Drafting the article: ET; 6. Revising the article critically for important intellectual content: AJ, RR, ZM, CN, IP, ME; 7. Final approval of the version submitted: all authors.

## Funding

The Swedish Research Council. Faculty of Medicine, Lund University, The Swedish Social Insurance Agency, Region Skåne and the County Councils of Southern Sweden.

## What this paper adds

Among patients in primary care the annual consultation prevalence has been reported to range from 2-10% of the population and incidence from 11-30/1000 person-years. However detailed information on for example age and sex characteristics and the number of repeat consultations is scarce.

An up-to-date Swedish estimate show an annual consultation prevalence of 1%, in men and women, and a new onset consultation rate of 80/10 000 women and 74/10 000 men.

Both measures increased by age up to retirement age, and then decreased.

## Pre-publication history

The pre-publication history for this paper can be accessed here:

http://www.biomedcentral.com/1471-2474/13/238/prepub
